# Supplementary material for: Loss of Foxc1 and Foxc2 function in chondroprogenitor cells disrupts endochondral ossification
Source: J Biol Chem. 2021 Jul 29;297(3):101020. doi: 10.1016/j.jbc.2021.101020 (PMC8383119; doi:10.1016/j.jbc.2021.101020)
Supplement: Table S3 [file mmc3.pdf]

Upregulated Genes Biological Processes	P-value
Peptide Cross-linking<br>*Crct1, Lce1a1, Lce1a2, Lce1b,Sprr2d*	3.1E-18
Keratinocyte Differentiation<br>*Crct1, Lce1a1, Lce1a2, Lce1b,Sprr2d*	2.8E-15
Lipid Metabolic Process<br>*Agpat5, Hmgcs2, Angptl8, Apod, Cpt1b, Cidea, Cyp11a1, Ephx2, Far2, Pnpla2*	9.0E-8
Epidermis Development<br>*Flg. Lce1f, Lce1h, Ptch2, Sprr2d*	2.8E-8
Oxidation-Reduction Process<br>*Ddo, Chdh, Cyp11a1, Far2, Fmo2, Sod3, Tryp1*	7.9E-4
Triglyceride Catabolic Process<br>*Lipe, Pnpla2, Pnpla3, Plin1*	8.7E-4
Regulation of Lipid Metabolic Process<br>*Angptl8, Hnf4a, Irs4, Pparg,*	3.8E-3
Brown Fat Cell Differentiation<br>*Adipoq, Fabp4, Mrap, Pparg*	4.1E-3
Negative Regulation of Smooth Muscle Cell Proliferation<br>*Ndrg2, Adipoq, Apod, Pparg*	5.7E-3
Positive Regulation of Fatty Acid Biosynthetic Process<br>*Mixipl, Agt, Hnf4a*	7.7E-3
